# A statistical approach to identify, monitor, and manage incomplete curated data sets

**DOI:** 10.1186/s12859-018-2121-6

**Published:** 2018-04-02

**Authors:** Douglas G. Howe

**Affiliations:** The Institute of Neuroscience, University of Oregon, Eugene, OR USA

**Keywords:** Zebrafish, *Danio rerio*, Gene expression, Machine learning, Curation

## Abstract

**Background:**

Many biological knowledge bases gather data through expert curation of published literature. High data volume, selective partial curation, delays in access, and publication of data prior to the ability to curate it can result in incomplete curation of published data. Knowing which data sets are incomplete and how incomplete they are remains a challenge. Awareness that a data set may be incomplete is important for proper interpretation, to avoiding flawed hypothesis generation, and can justify further exploration of published literature for additional relevant data. Computational methods to assess data set completeness are needed. One such method is presented here.

**Results:**

In this work, a multivariate linear regression model was used to identify genes in the Zebrafish Information Network (ZFIN) Database having incomplete curated gene expression data sets. Starting with 36,655 gene records from ZFIN, data aggregation, cleansing, and filtering reduced the set to 9870 gene records suitable for training and testing the model to predict the number of expression experiments per gene. Feature engineering and selection identified the following predictive variables: the number of journal publications; the number of journal publications already attributed for gene expression annotation; the percent of journal publications already attributed for expression data; the gene symbol; and the number of transgenic constructs associated with each gene. Twenty-five percent of the gene records (2483 genes) were used to train the model. The remaining 7387 genes were used to test the model. One hundred and twenty-two and 165 of the 7387 tested genes were identified as missing expression annotations based on their residuals being outside the model lower or upper 95% confidence interval respectively. The model had precision of 0.97 and recall of 0.71 at the negative 95% confidence interval and precision of 0.76 and recall of 0.73 at the positive 95% confidence interval.

**Conclusions:**

This method can be used to identify data sets that are incompletely curated, as demonstrated using the gene expression data set from ZFIN. This information can help both database resources and data consumers gauge when it may be useful to look further for published data to augment the existing expertly curated information.

**Electronic supplementary material:**

The online version of this article (10.1186/s12859-018-2121-6) contains supplementary material, which is available to authorized users.

## Background

In recent years, the biological sciences have benefited immensely from new technologies and methods in both biological research and computer sciences. Together these advances have produced a surge of new data. Biological research now relies heavily upon expertly curated database resources for rapid assessment of current knowledge on many topics. Management, organization, standardization, quality control, and crosslinking of data are among the important tasks these resources provide. It is commonplace today for these data to be widely shared and combined, increasing the impact that incomplete or incorrect data may have on downstream data consumers. Although assessing how complete or correct a large data set may be remains a challenge, examples have been reported. Examples include computational methods for identifying data updates and artifacts that may be of interest to downstream data consumers [1], machine learning methods to identify incorrectly classified G-protein coupled receptors [2], and to improve the quality of large data sets prior to quantitative structure-activity relationship modeling [3]. The completeness and quality of curated nanomaterial data has also been explored [4].

What does it mean for a data set to be “complete” or “incomplete”? Data can be incomplete in two ways: missing values for variables, or missing entire records which could be included in a data set. Handling missing variable values in statistical analyses is a complex topic outside the scope of this article. In the context of this work, “complete” means all currently published data of a specific type is present in the data set with no missing values for any variables. In this study, data from the ZFIN Database has been used to find genes that have an incomplete gene expression data set, genes for which there exist published but not yet curated gene expression data.

There are several reasons data repositories may not include all relevant published data, including high data volume, selective partial curation, delays in data access, and release of data prior to the ability to curate it. High data volume can result in the need for prioritization of the incoming data stream. For example, ZFIN is the central data repository for expertly curated genetic and genomic data generated using the zebrafish (*Danio rerio*) as a model system [5]. One major data input to ZFIN is the published scientific literature. A search of PubMed for all zebrafish literature shows that this corpus has consistently increased in volume by 10% every year since 1996 resulting in a greater than 10-fold increase in the number of publications processed by ZFIN in 2016 (2865 publications) compared to 1996 . Such increases necessitate prioritization to focus effort on the data deemed most valuable by the research community. As a result, curation of some publications is delayed or prevented all together. ZFIN currently includes curated data from approximately 25% of the incoming literature within 6 months of publication.

Data sets can also be incomplete relative to what has been published if publications are curated for selective data types. Publications that are not fully curated when they initially enter a database may later be partially curated during projects focusing on specific topics. For example, the gene functions were curated from all the publications associated with genes involved in kidney development [6]. In such cases, publications may get functional data, but no other data types, curated.

Delayed data access also contributes to curated data sets being incomplete. There is significant variation in how soon the full text of a publication may be available. Some journals have embargo periods which restrict publication access to those with personal or institutional subscriptions. Delayed access to the full text of publications slows data entry into data repositories, such as ZFIN, which require the full text to curate. ZFIN currently obtains full text for approximately 50%, 80%, and 90% of the zebrafish literature within 6 months, 1 year, and 3 years of publication respectively.

Incompletely curated data sets also result when new data types are published prior to database resources having the ability to curate those data. Curation of gene expression data commenced at ZFIN in 2005 [7]. Curating papers from earlier years, known as “back curation”, is something that many curation teams don’t have resources to support. Gene expression data published earlier than 2005 may only be curated at ZFIN if they were brought forward as part of an ongoing project or topic focused curation effort in subsequent years.

Why is it important to know if a data set includes all relevant published data? This knowledge can help database resources focus expert curation effort where it is needed. Likewise, if a researcher is aware that a data set may be missing records, they may look further for additional relevant published data to complete the data set. Having knowledge of all the published data helps to avoid wasted time, money, and effort repeating work already done by others, and also helps to avoid flawed hypothesis generation based on incomplete data.

In recent years, natural language processing (NLP) and machine learning methods have been widely used in the field of genetics and genomics on tasks such as prediction of intron/exon structure, protein binding sites, gene expression, gene interactions, and gene function [8]. In addition, model organism databases have used NLP and machine learning methods for over a decade to manage and automate processing of the increasing volume of publications that must be identified, prioritized, indexed, and curated [9–13]. These methods are applied to the incoming literature stream, prior to curation. Machine learning methods can also have utility after curation in maintaining the quality and completeness of curated data sets. The aim of this study was to provide a statistical approach to identify curated data sets that may be incomplete relative to what has been published. The ZFIN gene expression data set was the use case for this study. Researchers and data management teams alike can use the output of this method to guide resource allocation, decision making, and interpretation of data sets with insight into whether additional data may be available to augment an expertly curated data set.

## Results

### ZFIN gene expression annotations

**Fig. 1:**
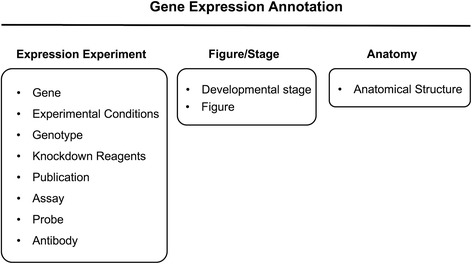
The structure of a gene expression annotation at ZFIN. Gene expression annotations at ZFIN are built up from three primary groupings of data: The expression experiment, the figure/developmental stage, and the anatomical structure. Each group of data contains specific pieces of information about the observed expression pattern

Gene expression annotations in ZFIN are assembled using a tripartite modular structure composed of: 1. The Expression Experiment; 2. The Figure number and Developmental Stage; and 3. the Anatomical Structure (Fig. 1). These modules are then combined to create each complete gene expression annotation. In this study, the count of gene expression experiments per gene was used as a key metric in the statistical model. The primary reason for this was largely a practical consideration. Due to the way the data are structured, the simplest approach was to count expression experiments per gene rather than to get a full count of complete gene expression annotations per gene.

### Descriptive analysis

**Fig. 2:**
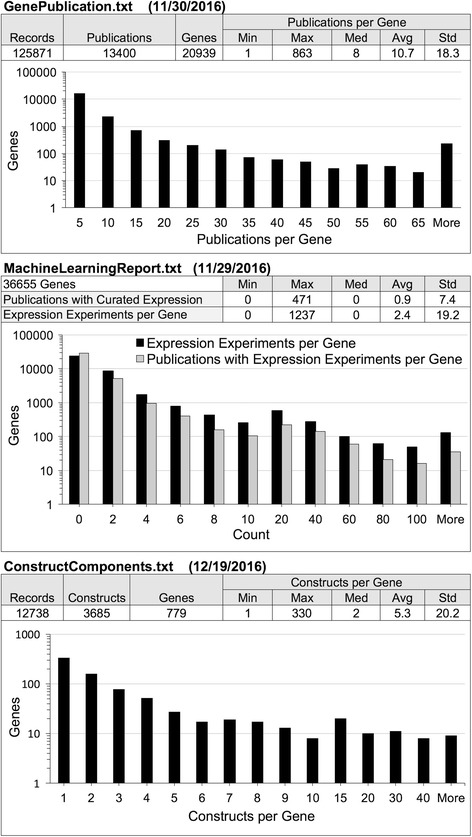
Descriptive statistics for the data used in model training and testing. Descriptive statistics are shown for the three data files used as input for training and testing the model: GenePublication.txt (top), ConstructComponents.txt (middle), and MachineLearningReport.txt (bottom)

Three data files were combined to build the predictive model. The MachineLearningReport.txt file included one row of data for each of the 36,655 gene records found in ZFIN at the time the file was generated. The GenePublication.txt file included 125,871 records describing which publications are attributed to which genes and what type of publications they are. ZFIN includes many publication types, but only journal publications were included in this study because they are the source of the gene expression annotations being modeled. The ConstructComponents.txt file included 12,738 records describing transgenic constructs and their components. Each construct that was related to a gene in this file was counted towards the number of constructs associated with a gene. There were 76 genes which had nine or more associated constructs, greater than 1.5 times the interquartile range for this data set. Summary statistics for these files are shown in Fig. 2.

### Feature selection

**Fig. 3:**
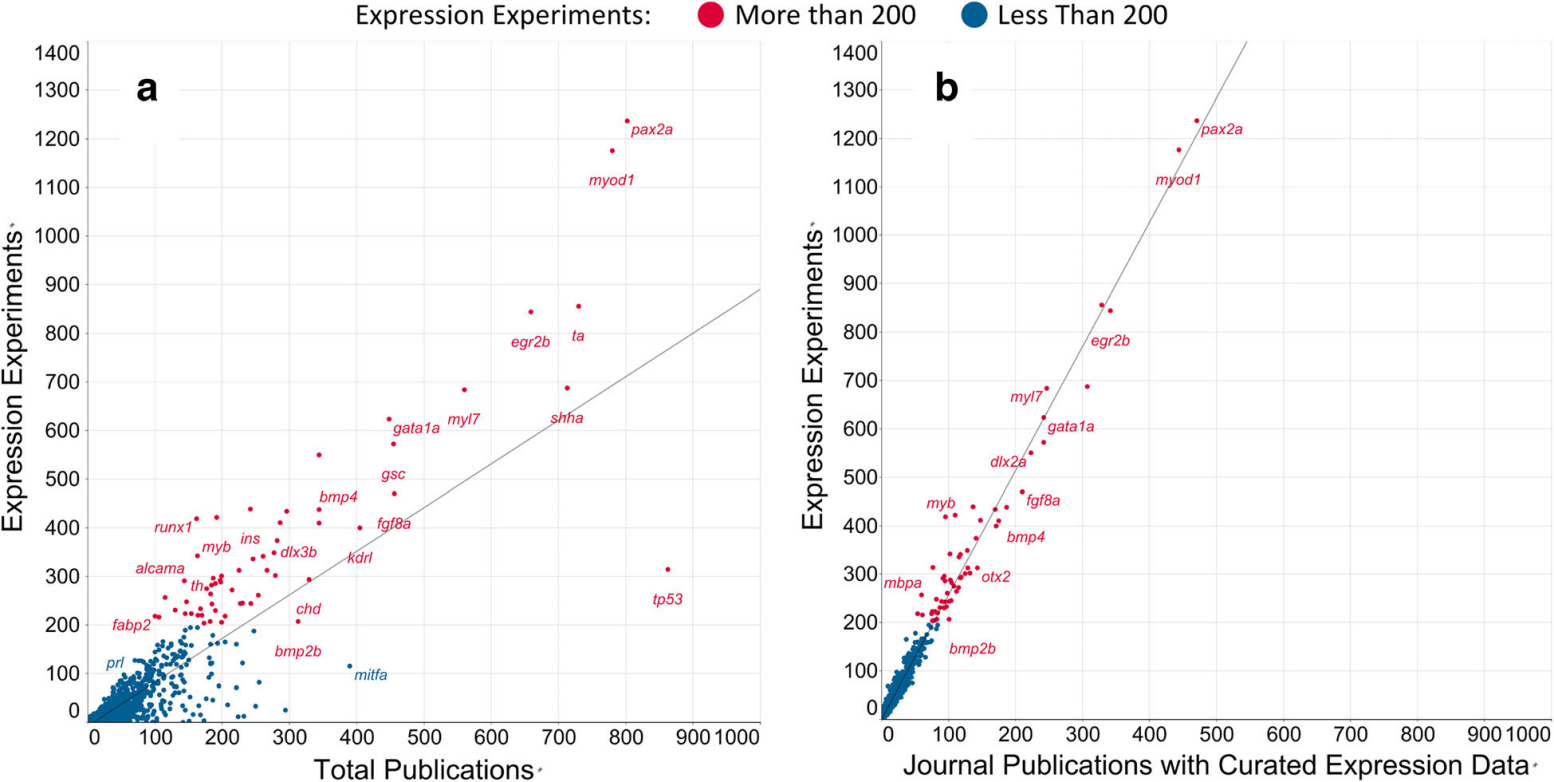
Correlation between the number of expression experiments and Publications. **a** The correlation between the number of expression experiments and the total number of journal publications per gene. **b** The correlation between the number of expression experiments and the number of journal publications having curated expression experiments per gene

Strong linear correlations were observed between the number of expression experiments per gene and the number of journal publications per gene (R^2^ = 0.82) or the number of journal publications curated for expression data per gene (R^2^ = 0.98; Fig. 3a and b respectively).

Of the 36,655 total gene records in this study, 12,851 had at least 1 expression experiment and averaged 6.8 (Std. Dev. = 32) expression experiments. These strong linear relationships between journal publications per gene and number of expression experiments per gene suggested that these variables could be the foundation of a linear model to predict how many expression experiments a gene should have. The higher correlation coefficient observed between expression experiments and journal publications curated for expression data (Fig. 3b) rather than total journal publications per gene (Fig. 3a) reveals that there are journal publications associated with genes for reasons other than gene expression, and that those are reducing the accuracy of the linear regression. For example, the locations of the *tp53* and *mitfa*, genes in Fig. 3a indicate that those genes have few expression experiments associated with them relative to the number of journal publications with which they are associated. Accounting for the additional reasons for associating a publication with a gene would strengthen the linear regression model. Additional variables were tested that could account for publications being associated with genes, including the number of Gene Ontology annotations and their associated journal publications, the number of phenotype annotations and their associated journal publications, and the number of transgenic constructs each gene was associated with. The model was trained and tested including these data, then the Azure Machine Learning (AML) Permutation Feature Importance module was used to evaluate their predictive value. Neither the phenotype annotation data nor the Gene Ontology annotation data provided any value towards prediction of the number of gene expression experiments per gene, so these were dropped from the model. The number of transgenic constructs and the gene symbol did have modest predictive value, so these were left in the model. The predictive value of the construct count is attributable to the 779 genes associated with one or more transgenic constructs, the maximum being 330 constructs associated with the gene *hsp70l.* The promoter of this gene has been used for nearly 20 years to drive inducible expression of transgenes with heat shock [14]. The addition of a feature for associated construct count per gene would make the model more accurate for those 779 genes to which this situation was applicable and hence improve model performance overall. The final list of features included in the model and their relative predictive value score as reported by the AML Permutation Feature Importance module is shown in Table 1.

**Table 1:** Variables selected for model training and their predictive value score.

Variable	Score
Journal publications with gene expression data	18.196293
Percent of journal publications with expression data	0.043752
Gene symbol	0.000215
Construct count	− 0.000447
Total journal publication count	−0.000692

### Regression modeling

Once the model variables were established, the input data set was split 25%/75% for model training and testing respectively of a linear regression model using the Azure Machine Learning Studio. The model was trained using the count of expression experiments as the label and set up to minimize the Root Mean Square Error (RMSE). The result of the predictive model run on the test data set was examined using the AML Evaluate Model module, which reported model performance results shown in Table 2. The high coefficient of determination (> 0.95) indicated that the model was a good predictor of the number of expression experiments per gene.

**Table 2:** Results of model testing

Measure	Value
Mean Absolute Error	1.582637
Root Mean Squared Error	3.368747
Relative Absolute Error	0.233714
Relative Squared Error	0.047532
Coefficient of Determination	0.952468

### Residual analysis

The purpose for making this model was to locate genes in the ZFIN database that have incompletely curated gene expression data sets relative to what has been published. The RMSE of the model output when run on the test data set was 3.368747 (Table 2).

**Fig. 4:**
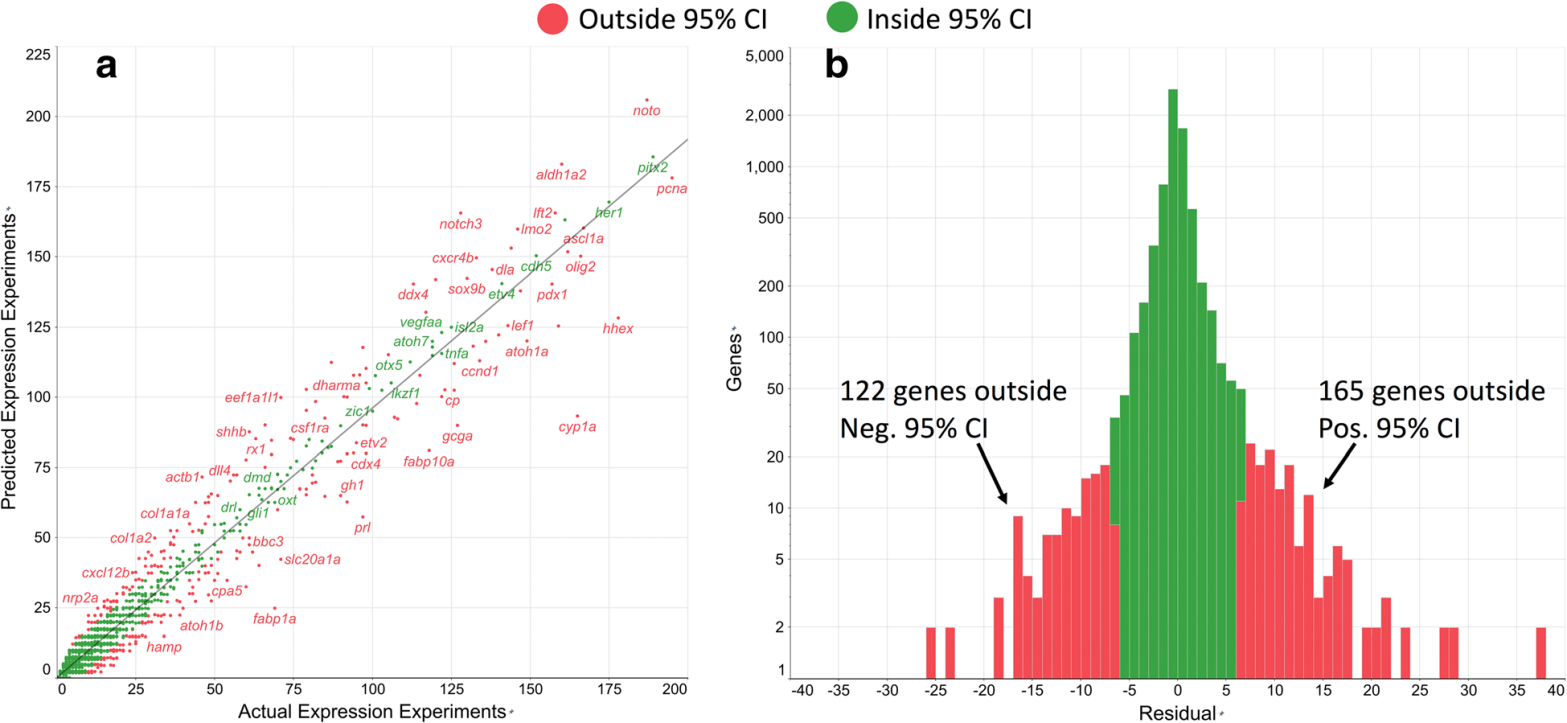
Actual vs. predicted expression experiment count per gene. **a**) Model predicted expression experiment count for the test data set was plotted against the actual expression experiment count per gene. A strong linear correlation was observed (R^2^ = 0.95), indicating that the model was accurate at predicting the number of expression experiments per gene. **b**) A histogram of expression experiment count residuals (actual number – predicted number) showed a single mode centered close to 0. Green and red bars are counts of genes inside or outside the 95% confidence interval respectively

Residuals were calculated as *y-f(x)* where *y* is the actual number of expression experiments per gene and *f(x)* is the model predicted number of expression experiments per gene. A scatter plot of *y* vs. *f(x)* produces a strong linear correlation (R^2^ = 0.95; Fig. 4a). The frequency histogram of these residuals reveals a single mode centered very close to 0, suggesting that the major variables for predicting expression experiment number per gene had been accounted for in this model (Fig. 4b). Genes with residuals that fell outside the 95% confidence interval (CI) of the model, calculated as two times the RMSE, were predicted to be missing published expression annotations. Of the 7387 genes in the test set 122 and 165 genes had negative or positive residuals respectively that were greater than twice the RMSE of the model and thus outside the model 95% confidence interval. Those genes were identified by this method as being significantly unlikely to have the complete set of gene expression experiments found in their associated journal publications.

### Model validation

**Fig. 5:**
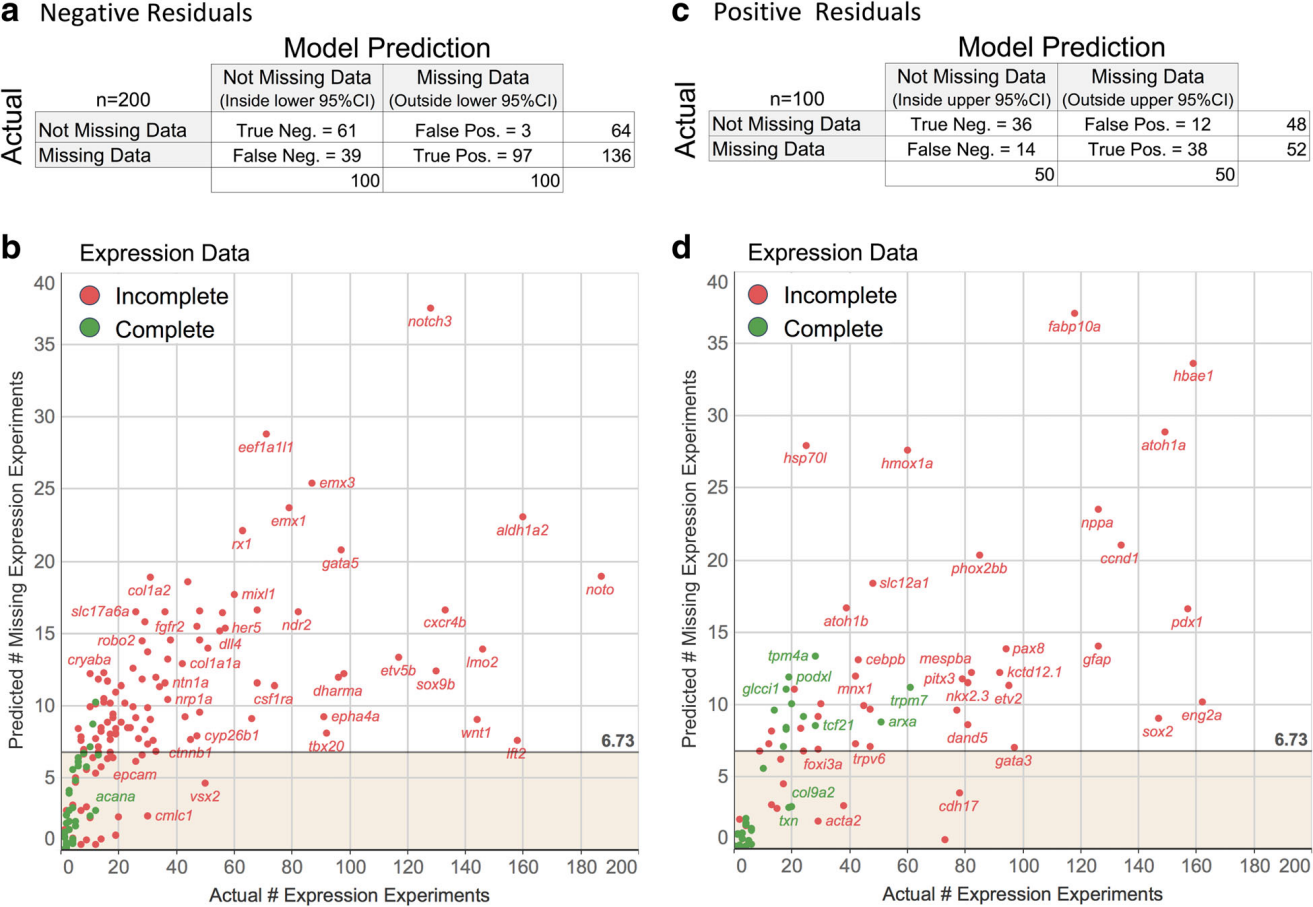
Quantification of model results. A confusion matrix of results from manual evaluation of model predictions for genes having negative (**a**) or positive (**c**) residuals. Columns show model predictions, rows show actual data status after manual validation. The actual expression experiment count was plotted against the predicted number of missing expression experiments per gene for each of the manually validated genes around the lower (**b**) and upper (**d**) 95% CIs. The horizontal line indicates the 95% confidence interval set at two times the root mean square error of the model. Genes above that line are predicted to be missing gene expression annotation, while genes below the line are predicted to not be missing gene expression annotations. Red dots are genes that were confirmed to be missing gene expression annotations. Green dots are genes that were confirmed to not be missing gene expression annotations

The model predictions were tested by manually examining journal publications associated with randomly selected genes from inside and outside the upper and lower 95% CI. One hundred genes having negative residuals were evaluated on each side of the lower 95% CI. Fifty genes having positive residuals were evaluated on each side of the upper 95% CI. For each gene, publications that had not already been curated for gene expression data were examined in chronological order starting with the earliest publication. The rationale for starting testing with the earliest publications was that publications from before 2005, when ZFIN started curating gene expression data, could be more likely to contain uncurated gene expression data. If that proved true, starting testing with the earliest publications could accelerate testing by identifying uncurated expression data in the older publications early in the testing process. After completing data validation, the count of genes having their earliest uncurated gene expression data per year was plotted. The data did not support the premise that earlier papers would be more likely to have uncurated expression data. Instead, a relatively random distribution was observed of earliest publication dates for the papers that had unannotated gene expression data (Additional file 1: Figure S1). These data are a complex function of multiple variables which change over time, including how many curators were working; the number, type, and timing of curation projects involving older literature; the number of other data types being curated concurrently; the volume of literature being managed; how curation priorities were set, etc. There are too many variables to draw any conclusions from this, other than that starting testing with the earliest publications was unlikely to have accelerated the testing process in this case. Genes were scored as having unannotated expression data as soon as one journal publication was found with unannotated expression data for that gene. Genes were scored as having a complete expression data set if all journal publications associated with the gene were examined and uncurated expression data for that gene was not found. Each of the manually validated genes having a negative residual or positive residual was plotted on a scatter plot of actual expression experiment count versus the number of expression experiments predicted to be missing (the residual) (Fig. 5b and d respectively). A line was drawn across the charts at 6.73 predicted missing expression experiments, which was the 95% confidence interval (2× RMSE) for the model. That line was used to separate genes predicted to be missing expression experiments (above the line) from those predicted to not be missing expression experiments (below the line). Red dots and green dots indicate genes that were or were not found to be missing gene expression annotations respectively during the validation. For genes having negative residuals, the model identified genes with published, but unannotated, expression data with a precision of 0.97 and recall of 0.71 (Fig. 5a). For genes having positive residuals, the model had a precision of 0.76 and recall of 0.73 (Fig. 5c). It is clear from this result that this method had high precision for finding genes in ZFIN that had published but yet to be curated gene expression data. This result also showed that the more expression experiments a gene had, the more likely it was to also be missing expression data. Additionally, there was a trend indicating that the higher the volume of existing expression experiments, the higher the number of predicted missing expression experiments was (Fig. 5b and d).

## Discussion

Expertly curated biological database resources contain highly accurate data [15]. Sometimes accuracy comes at the expense of being comprehensive due to prioritization of resource utilization, delayed data access, or publication of data that pre-dates its ability to be stored in a knowledge base. Efficient methods for identifying areas where data have been published but not yet curated are important for curators of data resources and users of those data resources alike. In this manuscript, the ZFIN gene expression data set was used as a test case to develop such a method. This method should be broadly applicable to *any* data set of sufficient size, as long as the proper predictive features can be identified. In the case of the ZFIN gene expression data, which has been captured from published literature by expert curators since 2005, the number of journal publications associated with a gene was an extremely good predictor of how many gene expression experiments a gene should have. This resulted in a simple linear model comprised of five variables. When the model was initially tested, genes associated with transgenic constructs were being reported with high significance as missing gene expression data, when in fact they were not. In some cases, genes associated with transgenic constructs had many dozens of publications associated with them which had no gene expression data for that gene. Perhaps the promoter of the gene was used in the construct for example, as is the case for the *hsp70l* gene. If that transgenic line was widely published, many publications ended up being associated with the gene because of the construct, even if there were no gene expression data for that gene in those publications. This led to the identification of the number of transgenic constructs per gene as an important variable in the model for those specific genes that were associated with constructs.

**Fig. 6:**
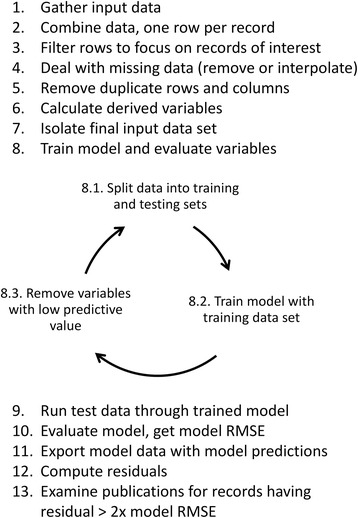
Generalized view of the method to find incomplete data sets

The process used here to identify incomplete curated gene expression data sets at ZFIN should be generalizable to other data sets, as summarized in Fig. 6. One key to extending this method to other data types is feature engineering, the use of domain knowledge to identify and craft variables having predictive value towards the desired variable. To find those predictive variables, data exploration and domain knowledge must first be applied to create a list of variables that may have predictive power. It is good to be inclusive at this point as it is not always possible to know which variables, variable derivatives, or variable interactions may be useful. In some cases data transformation, normalization, or computed values, such as the number of days since a record was last edited, may hold predictive value. There are many feature engineering techniques outside the scope of this paper which may be helpful in preparing data from other data sets. Once the data are assembled, the model training and testing process provides measures of model accuracy and predictive power of each variable. The best model will typically be the simplest model that still accurately represents the data. Variables that exhibit low predictive power should be dropped and the model re-trained and tested. This feature engineering, training and testing process is repeated iteraitively until model performance is acceptable and further removal of variables degrades model performance. A linear model was a good fit for the data examined in this work, but other data sets may be better fit using other types of models. The residual histogram provides one way to evaluate whether significant variables remain unaccounted for. Models with adequate variable representation have a bell shaped residual histogram centered around zero. If the histogram shape has “shoulders” or appears multi-modal, this is an indication that one or more variables are yet to be accounted for in the model, suggesting that the further feature selection and engineering could improve the model.

At ZFIN, every incoming zebrafish publication is associated with the genes discussed, even though not all the publications are fully curated. Hence, in ZFIN, the complete available literature across all genes is well represented, and thus the volume of published literature about a gene has a positive correlation with the amount of published data which exists for a gene. However, not all biological knowledgebases gather data using the same strategies. The method described here may not work as well for datasets that have more heterogenous representation of the published literature or other key variable. For example, a database which is populated with data by searching the literature for information about a specific record (gene, protein, etc.) may have deep representation of existing literature on the subset of records which have been researched and shallow representation of existing literature on other records. Heterogeneity of literature coverage of this type would detract from the predictive value of pure literature counts as were used for the ZFIN example. In such cases, other types of predictive variables would need to be identified through data exploration and feature engineering. These may include things such as the number of days since the last record update, number of data types associated, the presence of publications in specific journals, and presence of other potentially correlated data types. In some cases, it may be helpful to bring in additional data from external sources that can be linked to the data being examined. For example, UniProt records may not be associated with the complete literature about a protein or the associated gene. UniProt data for zebrafish proteins could be combined with the literature set from ZFIN for each related gene. This may increase the predictive value of the count of publications for identifying protein records in UniProt that are missing a piece of data of interest. Creative variable engineering will always be a critical step in successful application of this method.

The method described here produces a binary classification of genes that are predicted to be or not to be missing expression data based on the residual values being inside or outside the 95% CI of the model. A binary classification model makes sense for this problem. Unlike a binary classification, regression models result in a real number prediction of the label, in this case the number of gene expression experiments per gene. The regression model has the added possibility of providing a quantitative metric whose magnitude may correlate with the level of incompleteness of the data set. Confirmation of that possibility will require significant effort which should be the subject of future work.

This method can provide curators with a list of genes having published gene expression data that is yet to be curated. Therefore, the high precision outcome is important as it ensures that curators spend time reviewing publications for genes that *are* missing data. The model resulted in a recall/sensitivity of 0.71 and 0.73 at the lower and upper 95% CI, meaning 71% and 73% of the genes that were confirmed to be missing gene expression data were identified. From the perspective of a data curator, modest recall is acceptable for this method because subsequent rounds of model training and testing could be executed to iteratively refine and complete the data set. Genes that were not identified as missing data in the initial round of training and testing would eventually be identified in subsequent cycles of training, testing, and data updating. From the perspective of a data consumer, it would be beneficial to correctly identify as many genes as possible which have incomplete gene expression data sets. If future work finds that the magnitude of the residuals correlates well with the amount of missing expression data, then the residual itself could be provided to downstream data consumers as a metric of data set completeness for every gene included in the test set.

Machine learning methods are having significant impact upon many areas of our experience as scientists. As the field of data science has matured, these methods have become powerful tools for analysis, interpretation, and utilization of the increasingly large and interrelated data sets available today including numeric, free text, and image data. This work provides a machine learning approach to monitor data set completeness. It is concluded that this method could be used to identify incomplete data sets of any type curated from published literature, assuming proper predictive variables can be identified to build an accurate model.

## Methods

### Method overview

**Fig. 7:**
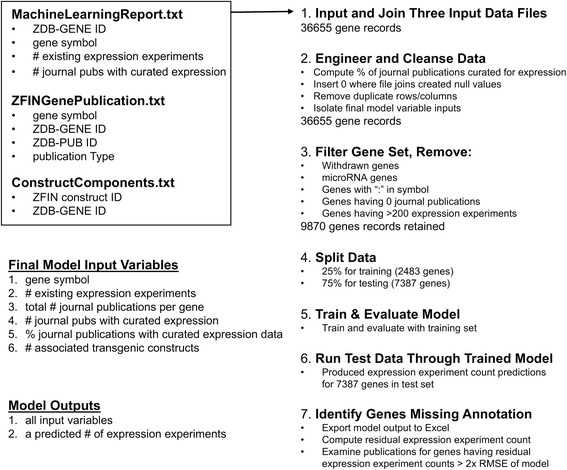
A summary of the method used to model the number of expression experiments per gene in the ZFIN gene expression data set

The method described here uses three data sets from the Zebrafish Information Network as input to a linear regression model to predict the number of gene expression experiments per gene. Figure 7 provides a flow chart of the steps taken from data input through model output.

### Data files

Three data files were combined to build the predictive model. All three are provided as supplementary files to this manuscript. The MachineLearningReport.txt file (Additional file 2) is a custom report consisting of one row per gene in the ZFIN database, generated on Nov. 29, 2016. Data columns included the ZDB-GENE ID, gene symbol, gene name, count of gene expression experiments, count of journal publications attributed for gene expression annotations, count of Gene Ontology annotations, and count of journal publications attributed for Gene Ontology annotations. The columns related to the Gene Ontology had no value for predicting the number of gene expression experiments, so they were excluded from further analysis.

The GenePublication.txt (Additional file 3) and ConstructComponents.txt (Additional file 4) files are generated daily at ZFIN and made available via the ZFIN downloads page (https://zfin.org/downloads). The GenePublication.txt file was obtained on Nov. 30, 2016. The columns were gene symbol, ZDB-GENE ID, ZDB-PUB ID, publication type, and PubMed ID when available. The ConstructComponents.txt file was obtained on Dec. 19, 2016 and included columns for the ZFIN construct ID, construct name, construct type, related gene ZDB-GENE ID, related gene symbol, related gene type, a relationship between the gene and the construct, and two ontology term IDs from the sequence ontology [16] to specify the type of construct and the type of related marker. For this study, the only data used was a count of constructs related to each gene, which was computed from the ConstructComponents.txt file.

### Data preparation and modeling

Manipulations of input data files, feature selection and engineering, model building, training, evaluation, model selection, and final model scoring were all done using modules provided in Microsoft Azure Machine Learning Studio (https://studio.azureml.net) using a free workspace level account. Features per gene used to train and test the linear regression model included the gene symbol, the number of journal publications attributed for gene expression, the number of gene expression experiments (the label), total number of journal publications, the percentage of journal publications with curated expression data, and the number of transgenic constructs associated with each gene.

The set of all gene records in the ZFIN database (36,655 genes as of Nov. 29, 2016) was filtered to exclude genes that were unlikely to be useful in this analysis including withdrawn genes, microRNA genes, genes with a colon in the name (typically not yet studied), genes with symbols starting with “unm_” (typically not yet studied), and genes with no associated journal publications as determined by data from the GenePublication.txt file. Genes with more than 200 existing expression experiments were also excluded because they are already heavily annotated for gene expression, many were found to be anatomical marker genes of less interest for the purposes of this work (eg. *egr2b)*, and their heavy annotation may give them undesirable leverage that could negatively affect model performance for genes of interest which may have few annotations. Those excluded genes having more than 200 expression experiments have red symbols in Fig. 3. The resulting gene set used as input for model training and testing included 9870 genes. Any null numeric values generated in the data during file joining were set to 0 using the AML Clean Missing Data module, and no duplicate rows were present. A stratified split keyed on the expression experiment count was used in the Split Data module to select 25% of the genes (2483 genes) for training the model and 75% (7387 genes) for scoring the model. The Linear Regression, Train Model, and Score Model modules were used to train and score the model. The Linear Regression module used the following parameters: Solution method: ordinary least squares; L2 regularization weight: 10; Include intercept term: unchecked; Allow unknown categorical levels: checked; Random number seed: 112. Model performance was assessed using the Azure Machine Learning Evaluate Model module. The trained model was used to predict the number of expression experiments for the 7387 genes that were not used in model training. The resulting prediction was appended as a new column to the input data set.

### Analysis and data visualizations

Model results, including the input data plus the predicted number of expression experiments, for the 7387 genes were exported from Azure Machine Learning Studio as a tab delimited file and imported into Microsoft Excel for Mac v16.27 for data validation and analyses (Additional file 5). Residuals were calculated as the actual expression experiment count minus the number of expression experiments predicted by the model. The 95% confidence interval of the model, computed as 2 times the root mean squared error (RMSE), was used to establish significance of the residuals. Genes with residuals outside or inside the 95% confidence interval were then considered as being predicted to be missing or not missing expression annotation respectively. One hundred genes inside and outside the negative 95%CI were randomly selected for manual testing by sorting the genes in the Excel spread sheet based on a randomly generated number column and copying the first genes from each set into a new Excel sheet. To remain blinded during the evaluation step, those genes were randomized again as a set by sorting based on a randomly generated number column. That gene selection process was repeated for 50 genes inside and outside the positive 95% CI. A manual evaluation was then done for each journal publication not already curated for expression data that was associated with each of the selected genes. The publications for each gene were sorted oldest to newest based on publication date and were then evaluated in order, starting with the oldest publications. Publication assessment for each gene continued until either all the publications were examined for a gene or a publication with missing expression data for that gene was identified, whichever came first. The result was recorded along with the assessment date and the ZDB-PUB ID for the publication that was missing the expression data, if one was found. The results of this data validation was used to produce a confusion matrix describing model precision and recall around the upper or lower 95% CI.

Publication records in ZFIN each have a unique ZDB-PUB ID, for example ZDB-PUB-161203-17. The first six digits indicate the date the record was created in YYMMDD format. Those data were parsed out of the list of IDs for publications that were recorded as containing uncurated gene expression data. The year component was then used to group those data to get a count of the number of genes per year that were found to have uncurated gene expression data. Even though it was the year of publication entry into ZFIN that was being counted, only the first paper encountered with uncurated expression data was recorded per gene, so the count is equal to the number of genes in the sample having uncurated expression data from each year.

Data visualizations were created using both Excel and Tableau Desktop Professional Edition v10.1.4.

## Additional files

Additional file 1: **Figure S1.** The count of genes having their earliest uncurated gene expression data published in each year. (PNG 7782 kb)

Additional file 2: The MachineLearningReport.txt file. (TXT 2317 kb)

Additional file 3: The GenePublication.txt file. (TXT 22006 kb)

Additional file 4: The ConstructComponents.txt file. (TXT 1807 kb)

Additional file 5: The Excel file which includes model results and data analysis results. (XLSX 8346 kb)
